# Distinguishing Genuine Imperial Qing Dynasty Porcelain from Ancient Replicas by On-Site Non-Invasive XRF and Raman Spectroscopy

**DOI:** 10.3390/ma15165747

**Published:** 2022-08-20

**Authors:** Philippe Colomban, Michele Gironda, Gulsu Simsek Franci, Pauline d’Abrigeon

**Affiliations:** 1 MONARIS UMR8233, Sorbonne Université, CNRS, 4 Place Jussieu, 75005 Paris, France; 2 XGLab S.R.L—Bruker, 23 Via Conte Rosso, 20134 Milan, Italy; 3 Surface Science and Technology Center (KUYTAM), College of Sciences, Rumelifeneri Campus, Koç University, 34450 Istanbul, Turkey or; 4 Musée des Arts d’Extrême-Orient, Fondation Baur, Rue Munier-Romilly 8, 1206 Geneva, Switzerland

**Keywords:** porcelain, imperial bowl, reign mark, color, pigments, elemental composition, cobalt, arsenic, gold nanoparticles, pyrochlore

## Abstract

The combined use of non-invasive on-site portable techniques, Raman microscopy, and X-ray fluorescence spectroscopy on seven imperial bowls and two decorated dishes, attributed to the reigns of the Kangxi, Yongzheng, Qianlong, and Daoguang emperors (Qing Dynasty), allows the identification of the coloring agents/opacifiers and composition types of the glazes and painted enamels. Particular attention is paid to the analysis of the elements used in the (blue) marks and those found in the blue, yellow, red, and honey/gilded backgrounds on which, or in reserve, a floral motif is principally drawn. The honey-colored background is made with gold nanoparticles associated with a lead- and arsenic-based flux. One of the red backgrounds is also based on gold nanoparticles, the second containing copper nanoparticles, both in lead-based silicate enamels like the blue and yellow backgrounds. Tin and arsenic are observed, but cassiterite (SnO_2_) is clearly observed in one of the painted decors (dish) and in A676 yellow, whereas lead (calcium/potassium) arsenate is identified in most of the enamels. Yellow color is achieved with Pb-Sn-Sb pyrochlore (Naples yellow) with various Sb contents, although green color is mainly based on lead-tin oxide mixed with blue enamel. The technical solutions appear very different from one object to another, which leads one to think that each bowl is really a unique object and not an item produced in small series. The visual examination of some marks shows that they were made in overglaze (A608, A616, A630, A672). It is obvious that different types of cobalt sources were used for the imprinting of the marks: cobalt rich in manganese for bowl A615 (Yongzheng reign), cobalt rich in arsenic for bowl A613 (but not the blue mark), cobalt with copper (A616), and cobalt rich in arsenic and copper (A672). Thus, we have a variety of cobalt sources/mixtures. The high purity of cobalt used for A677 bowl indicates a production after ~1830–1850.

## 1. Introduction

It is well established that Chinese antiquarianism became really systematic in terms of the collection and study of ancient relics during the Song dynasty onward [1,2]. The interest of the Chinese elite in objects from remote dynasties is also well established for bronzes [3,4] as well as for glazed ceramics [5,6,7,8,9,10,11]. In addition, the aura given to an object in Asia is primarily a function of the social status of its owner [12]. Consequently, objects manufactured for the emperor, in particular those produced in the imperial workshops, are, therefore, particularly prized. The European elites discovered the production of Chinese and then Japanese porcelains with the establishment of regular Portuguese maritime relations in the 16th century. It was especially in the 17th and 18th centuries when imports developed on a large scale, mainly via the Dutch and English companies and, to a lesser extent, French or Swedish. Collections of oriental objects started and, therewith, the commercial activity of imported ‘old’ or contemporary objects, adapted to the clientele’s tastes, in particular in France by the *‘marchands-merciers*’, who altered artifacts to suit local taste by adding gilded bronze [13,14,15]. The Universal Exhibitions organized from the middle of the 19th century [16,17] expanded the interest shown in Asian porcelains and the number of ‘connoisseurs’ collecting these objects [18,19]. The forced opening of China by the Western powers and events such as the sack of the Summer Palace led to the arrival on the market of objects from the imperial collections in particular ‘imperial’ ones [20,21,22,23,24,25]. One of the Universal Exhibitions’ objectives was to identify and show manufacturing techniques to help assimilate new know-how and produce new products. This led to making replicas. ‘Engineer-artists’, such as Théodore Deck [26,27,28,29], and engineer-managers, such as Alexandre Brongniart [30,31,32] for ceramics and Philippe-Joseph Brocard [33] for glass, devoted their activities to replicating the great masterpieces of other civilizations and creating objects freely inspired by models. Some entrepreneurs, such as the Manufacture Samson [34,35,36], devoted their activity to making high-quality replicas. Today, the art market encounters many fakes of various qualities. Developing objective analytical tools for identification, in addition to a subjective visual expertise, is, therefore, not only a real need but also a challenge.

It is important to recall that, for a very long time, the Arts of Fire only used natural raw materials, visually selected, ‘purified’ by simple operations (grinding, washing, heat treatment) [37,38,39,40], both for the products of the base—powdered rocks, predominant by weight (clays, sands, and feldspars and, eventually, grog)—and the materials used in much smaller quantities for enamels, which constitute only around 1 %wt or less of the object. Coloring agents themselves are a very minor part of the enamel, from 0.5 to 5 %wt oxide [40,41,42,43], and raw materials providing color were traded over long distances. The composition of the raw materials is, therefore, not constant, and the variability depends on the visual selection, natural solid solutions, and impurities of the minerals constituting the ores used and their ‘purification’ treatment. During the 19th century, mainly in the second half, the more or less ‘purified’ natural coloring products were replaced by ‘chemicals’, namely, salts (carbonates, sulfates, nitrates) or oxides with a much lower number of impurities [44,45,46,47,48,49,50,51,52]. Therefore, identifying the elements associated with those used to color enamels can be an effective authentication tool [50,51,52].

It is important to differentiate a genuine artifact attesting to an esthetic innovation from ‘replicas’ or ‘copies’ or ‘fakes’ (it is necessary to identify the motivation to use the right qualifier) [53,54]. On the other hand, it is common for an esthetic line to continue to determine shapes and decorations long after its appearance. The identification of specific raw materials used in the artifacts is now possible on site with two non-invasive methods: Raman microspectroscopy and X-ray fluorescence spectroscopy. We present here the first analysis of seven exceptional enameled bowls of the *huafalang* 畫琺瑯 or *falangcai* 琺瑯彩 type (i.e., word-to-word made with Western colors), bearing the imperial mark of the various reigns of the Qing Dynasty, from the collection of the Baur Foundation, in addition to two dishes of the same periods. The studied objects are high-grade examples of 18th century work, such that their qualities are equivalent to oil and pastel paintings, like previous productions of majolica, enamels of Limoges, and enameled watches [55,56]. The three-dimensional heterogeneity of the colored zones led us to compare the data on the number of photons by looking at the ratios of elements which appeared to us, by reasoning, to be relevant. The priceless values of studied artifacts act against their displacement to the laboratory, and also, a fortiori, sampling is prohibited. Comparison with significant series of previous data recorded with different instruments was used to support the discussion after normalization of the data.

To date, only three of these exceptional imperial bowls have been analyzed at the National Museum of Asian Arts-Guimet (Paris, France) but only with Raman microscopy [44]. Elemental composition ratios of blue areas of these bowls have also been compared with those of artifacts assumed to have been made at the Custom District of Guangzhou (Canton), in a recent paper [52]. Rare shards from similar artifacts have also been analyzed [45,46,47,48]. Results will be discussed in order to identify the possible replicas.

## 2. Materials and Methods

### 2.1. Artifacts

**Figure 1:**
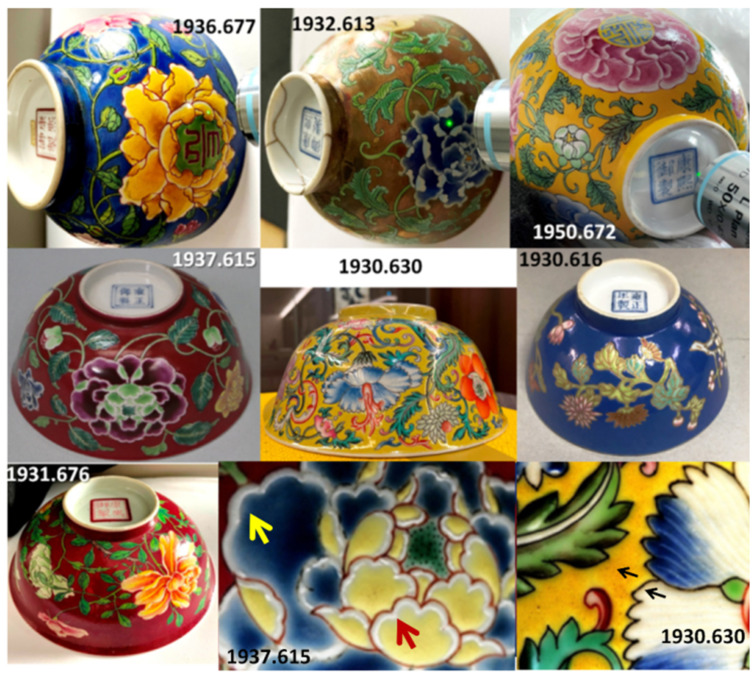
Views of the imperial bowls. The zooms show the extra thickness deposit (yellow and red arrows), the black lines separating the colored areas, and the heterogeneity of the colored areas (black arrows). See Table 1 for more information. For simplification, only the last three digits of the inventory number (after the point) are used in the text following the letter A.

**Figure 2:**
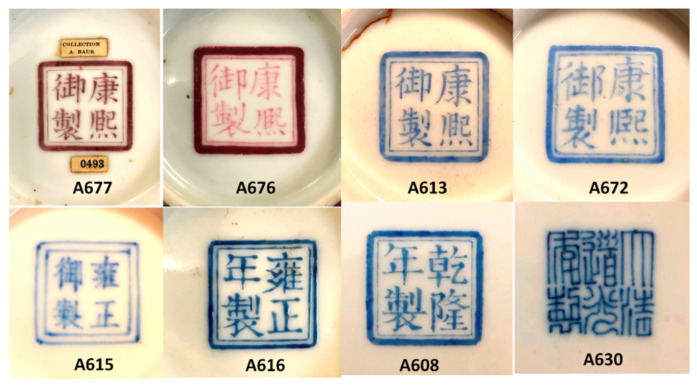
Views of the imperial bowl marks. See Table 1 for details.

**Figure 3:**
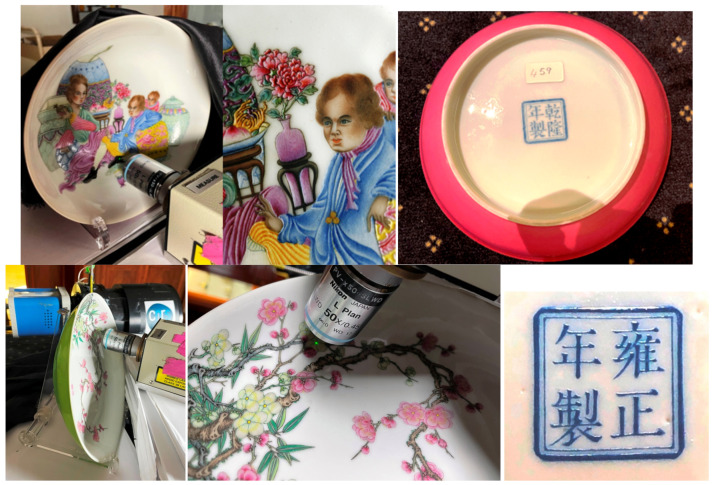
Views of the two dishes under Raman examination (top A608, bottom A596, see Table 1).

Figure 1 presents the studied imperial bowls. Marks are shown in Figure 2, and two dishes are presented in Figure 3. The dating attributions, dimensions, and areas analyzed are listed in Table 1.

The floral decoration of the bowls (Figure 1) is painted ‘on’ (or left in reserve) a plain background, which is colored in yellow (A672, A630), honey-gold (A613), red (A615, A676), and blue (A677, A616). The reign marks (Kangxi, Yongzheng, and Daoguang) are colored either in blue (A613, A672, A615, A630, A616) or red (A677) (Figure 2). A black line is usually used to separate colored areas, as made by Ottoman potters [29,57]. The extra thickness of certain colors is obvious, which indicates their subsequent deposit by additional firing, for example, for the white border of the flower of A615 shown in Figure 1. At the (sub)millimeter scale, visual observation identifies the heterogeneity of the colors, for example, for the yellow and green areas of bowl A630. All of the porcelain bodies are very white. The thicknesses of the enamels look variable, the thinner ones being observed for the dish decoration (e.g., Figure 2), except for a few colors (pink of the flowers in the bouquet of A608, Figure 3).

The selection of objects includes imperial bowls bearing a Kangxi *yu zhi* 康熙御製 mark (lit. “made by imperial command of the Kangxi emperor” r. 1662–1722) (A613, A672, A676, and A677) supposed to be painted in the Palace workshops (Zaobanchu, 造辦處) in Beijing, in particular, a bowl (from a pair) with a gilded background and a flower and floral scroll design in polychrome enamels on the glaze (A613b) and a yellow-bottomed bowl with similar decoration but where the pistil of the flowers is decorated with auspicious characters (A672). These last two artifacts show the Kangxi *yu zhi* mark (in overglaze?) in cobalt blue on the base (Figure 2). At the time of the first publication of these objects, there was a controversy in Western academic circles questioning the production of *Famille rose* enamels, i.e., opaque enamels dominated by the use of a pink glaze obtained by means of colloidal gold, for the Kangxi period [58], such that the objects were at first considered of later date [59] before being reattributed to the Kangxi period [60]. The publication of the Chinese Imperial Collections has helped to dispel the doubts expressed by these specialists. However, apart from these three pieces, which seem to belong to the Kangxi period, the Baur Foundation also possesses two bowls (A676 and A677) also bearing a Kangxi period mark (this time in red on the glaze, Figure 2) whose style differs clearly from this first series. Artifact A677, a bowl with a blue background, shows a decoration of leaf and flower scrolls figured with the idiom *wan shou chang chun* 萬壽長春 (meaning “a myriad of happiness and longevity”), and the shape evokes a pair currently preserved at the British Museum in London (inv. 1936,0413.33 [61]). The bowl of the Baur Foundation is, however, far from equaling in finesse the decoration of the above-mentioned pair: the overglazed enamels look thicker, the gradation between the colors much less subtle, and the veins of the leaves are reduced to a few strokes, which explains why doubts persisted as to its attribution. On the other hand, the A676 bowl presents an unusual decoration for the period: the flowers, instead of being arranged in a hieratic way at regular intervals on both sides of the bowl, are intermingled and seem to swirl around the bowl. The treatment of the flowers leaves an important part to the nuances of color, giving relief and depth to each petal. The atypical character of the decoration allows us to question the date of the piece.

The objects of the Yongzheng reign 雍正 (1722–1735) include a bowl on a red background (A615) in the continuity of those mentioned above but where the mark (Yongzheng *yu zhi*) is this time applied with cobalt blue underglaze, which would correspond to a production of Jingdezhen (usually called *yangcai* 洋彩, pieces made and painted at Jingdezhen) [62,63]. In addition, there is a small bowl (A615) with a blue background and a decoration outlined in gold, reminiscent of the *cloisonné* enamel technique. Here again, the atypical character of both the shape and the decoration of the piece made attribution difficult. Finally, a dish (A596) decorated with a poem and prunus in flower and with a lime green reverse—one of the new colors created at the turn of the 18th century in China—completes this set [64].

For the Qianlong reign 乾隆 (1736–1795), only one emblematic piece (A608, Figure 3) was chosen: a dish with a red reverse side and a European character decoration, a type of decoration that appeared during this period.

Finally, as a comparison, and to observe a possible evolution in the composition of enamels in the 19th century, an imperial bowl from the Daoguang 道光 reign (1821–1850) with a yellow background and a decoration of intermingled floral scrolls was chosen. This decoration imitates a pattern already visible during the Qianlong period (see similar bowl with Qianlong mark, British Museum, inv. Number Franks.577.+) [65].

### 2.2. Methods

#### 2.2.1. Portable X-ray Fluorescence Spectroscopy (pXRF)

X-ray fluorescence analysis was performed on site using a portable ELIO (XGLa Bruker, Berlin, Germany) instrument as in previous studies [52,55,56,66]. The set-up included a miniature X-ray tube system with a Rh anode (max voltage of 50 kV, max current of 0.2 mA, and a 1 mm^2^ collimator) and a large-area Silicon Drift Detector (SDD, 50 mm^2^ active areas) (XGLab Bruker, Berlin, Germany) with an energy resolution of <140 eV for Mn Kα, an energy range of detection from 0.9 keV to 42 keV (from 1.3 keV in air), and a maximum count rate of 5.6 × 10^5^ cps. Depending on the object, the measurement was carried out by positioning the instrument on the top or on the side. Perfect perpendicularity to the area measured is needed.

Measurements were carried out in the point mode with an acquisition time of 120 s, using a tube voltage of 50 kV and a current of 80 μA. No filter was used between the X-ray tube and the sample. During the analysis, the working distance between the sample and detector was around 15 mm, and the distance between the instrument front and artifact was about 10 mm. Spectral signals were obtained with the optimization of the signal-to-noise ratio (SNR) by selecting the set-up parameters chosen. The analysis depth during the measurement of the enamel was estimated from the Beer–Lambert law (analysis depth, defined as the thickness of the top layer from which comes 90% of the fluorescence) to be close to 6 µm at Si K_α_, 170 µm at Cu K_α_, 300 µm at Au L_α_, and 3 mm at Sn K_α_. Within the resolution of the pXRF instrument, the Fe K_β_ peak, which may refer to the red pigment, and the Co K_α_ peak corresponding to the blue color are located in the same energy range. To visually identify the presence of cobalt in the enamel spectrum (except when cobalt is present in traces), we can use the information obtained from looking at the Fe K_α_/Fe K_β_ ratios. In the absence of cobalt, the relative intensity between Fe K_α_ and Fe K_β_ peaks is about 6/1 [52]. Cobalt is then obvious if the superimposed peaks of Co K_α_ and Fe K_β_ exhibit a stronger intensity than that expected from the above ratio.

#### 2.2.2. Processing of XRF Data

**Figure 4:**
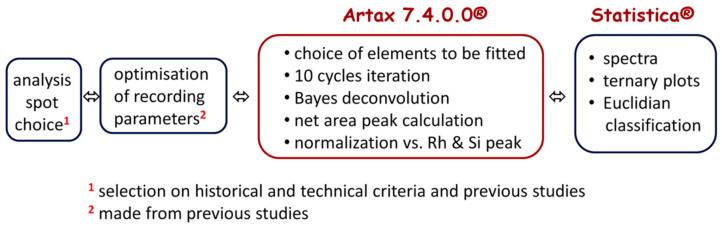
Flowchart of the XRF study and data evaluation procedure.

Figure 4 shows the flowchart of the procedure. After recording the raw data with ELIO, the Spectra (the so-called .spx) files were opened in the Artax 7.4.0.0 (Bruker, AXS GmbH, Karlsruhe, Germany) software. For the data treatment process, the studied objects were considered infinitely thick samples. Before evaluating the analysis data, all of the spectra were imported, and a new method file was created via “Method Editor” of Artax for an applied voltage of 50 kV and current of 80 μA. The corresponding major (e.g., K, Ca), minor (e.g., Fe, Ti, Co), and trace elements (e.g., Ag, Bi, As) were added to the Periodic Table. For the correction, escape and background options were selected in the Method Editor, and 10 cycles of iteration were selected starting from 0.5 keV to 45 keV. The deconvolution method, Bayes, was applied to export the data table. The net area was calculated under the peak at the characteristic energy of each element selected in the periodic table, and the counts of the major, minor, and trace elements were determined in the colored areas (white, red, yellow, orange, blue, green, and black). A normalization with respect to the signal Si was made for the comparison of certain elements, in particular for the data coming from different measurement campaigns. Before plotting the scatter diagrams, the net areas of each element were normalized by the number of XRF photons derived from the elastic peak of the X-ray tube of rhodium. Then, these normalized data were plotted in the ternary scattering plots and tree clustering plots drawn for the interpretation and discussion of the results with the software Statistica 13.5.0.17 (TIBCO Software Inc., Palo Alto, CA, USA).

#### 2.2.3. Raman Microspectroscopy

Raman analyses were carried out in the museum exhibition room (Figure 2) with a mobile HE532 Raman set-up (HORIBA Scientific Jobin-Yvon, Longjumeau, France) as extensively described in the references [55,56,66,67]. For each colored area in the objects, at least three Raman spectra were recorded to control the representativeness of the collected data on a statistical basis. The reliability of the Raman spectrum starts above 80 cm^−1^, but a flat spectral background is only obtained over 500 cm^−1^. A 50× (17 mm long working distance, Nikon France SAS, Champigny-sur-Marne, France) objective was used (surface spot size ~2–4 µm; in-depth ~5–10 µm, the values varying with the color), perpendicular to the sample surface, which allowed the recording of spectra not/poorly contaminated by the sub-layers and/or the silicate matrix if grains were bigger than ~5 µm. Obviously, the power of illumination at the sample should be minimal (1 mW) for dark-colored areas, due to the absorption of light, although up to 10 mW is required for light-colored enamels and more for body and colorless glaze. Unfortunately, measurements performed on site require a rather high power of illumination that can induce phase transformation and oxidation of absorbing phases (dark-colored or black).

## 3. Results

**Figure 5:**
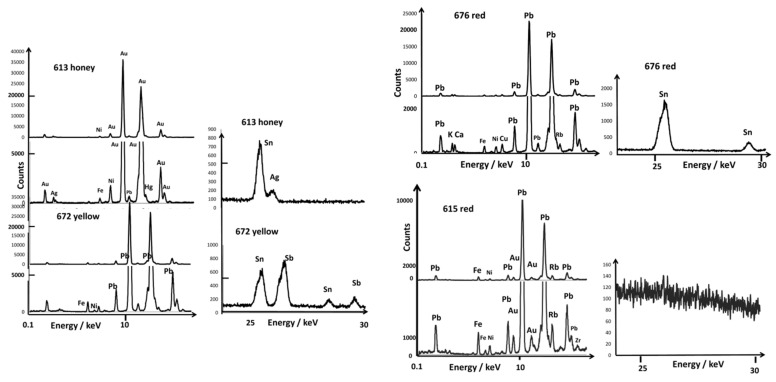

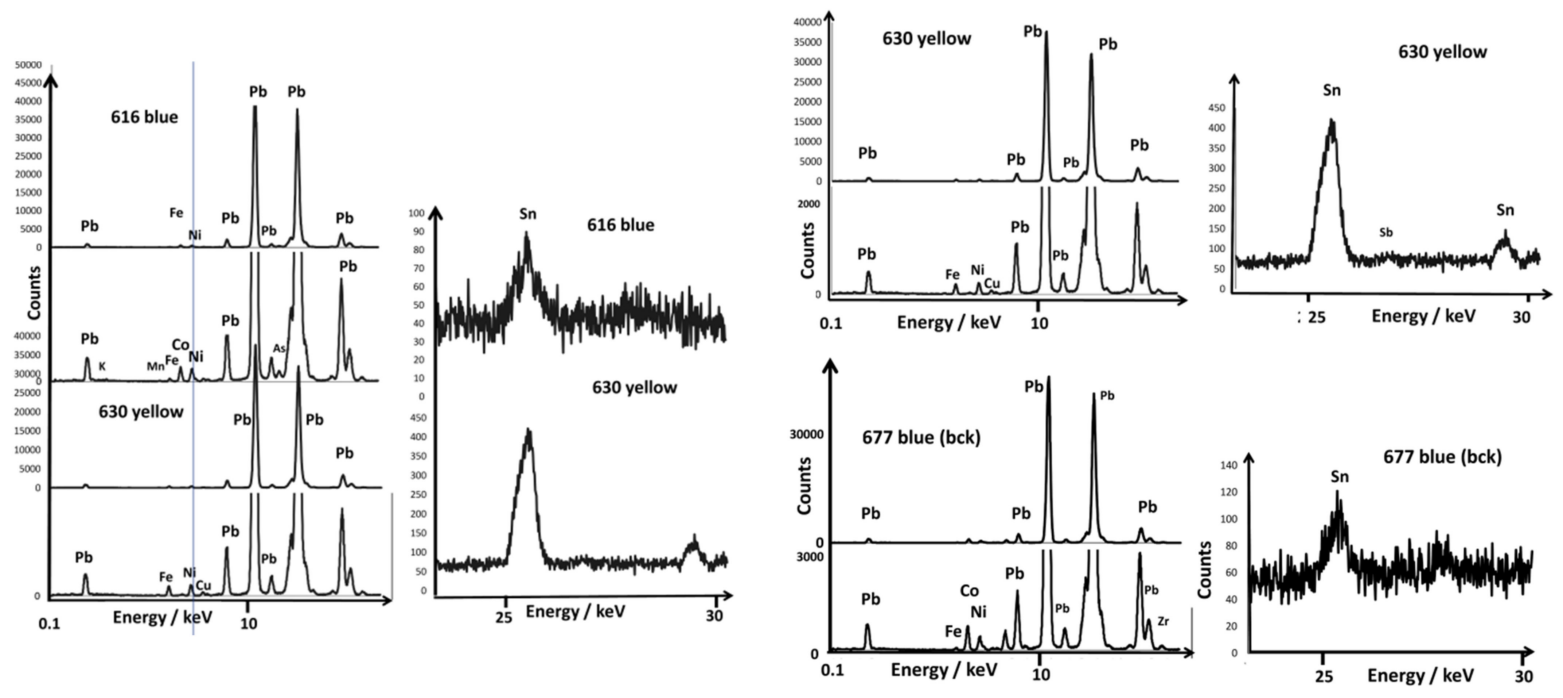
Comparison of the representative XRF spectra for yellow, golden honey, blue, and red backgrounds recorded in the 0.1–20 and 20–30 keV energy ranges; for comparison, some spectra of paste are also given. Line is guide for eyes.

**Figure 6:**
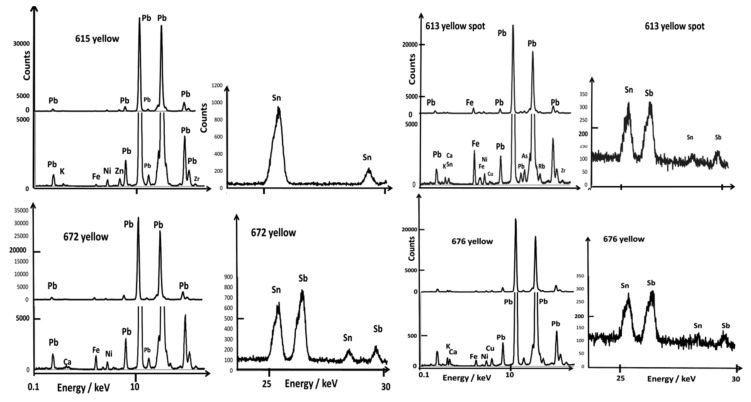
Comparison of representative XRF spectra for yellow painted decor in the 0.1–20 and 20–30 keV energy ranges.

In this section, we will first visually examine the XRF spectra of the enamels/glazes and colored areas. Then, to compare the data, we will use our net XRF photon counts comparison approach [52] through ternary diagrams concerning the relevant elements and compare the enameling and coloring technologies by defining the characteristic elements of fluxes, coloring agents, and associated impurities. It is important to keep in mind that, due to the nature of the X-ray–matter interaction phenomenon, XRF intensities—visually—are not directly representative of the composition in the volume analyzed, which is highly variable throughout the energy of the X-ray photons represented by the horizontal axis (Figure 5). Thus, in the spectrum of the body in bowl A615, although the element silicon is the main element, its peak (transition Kα) is weak. Moreover, the Kα peak of iron (and Kβ much weaker), for instance, appears stronger, whereas the proportion of this element is at least ten times lower than that of silicon. A small peak of manganese and traces of nickel, copper, zinc, titanium, yttrium, and zirconium are also visible. The XRF spectra of the glazes are very similar, except that the calcium peaks (Kα and Kβ) are a little more intense (Figure 6).

**Figure 7:**
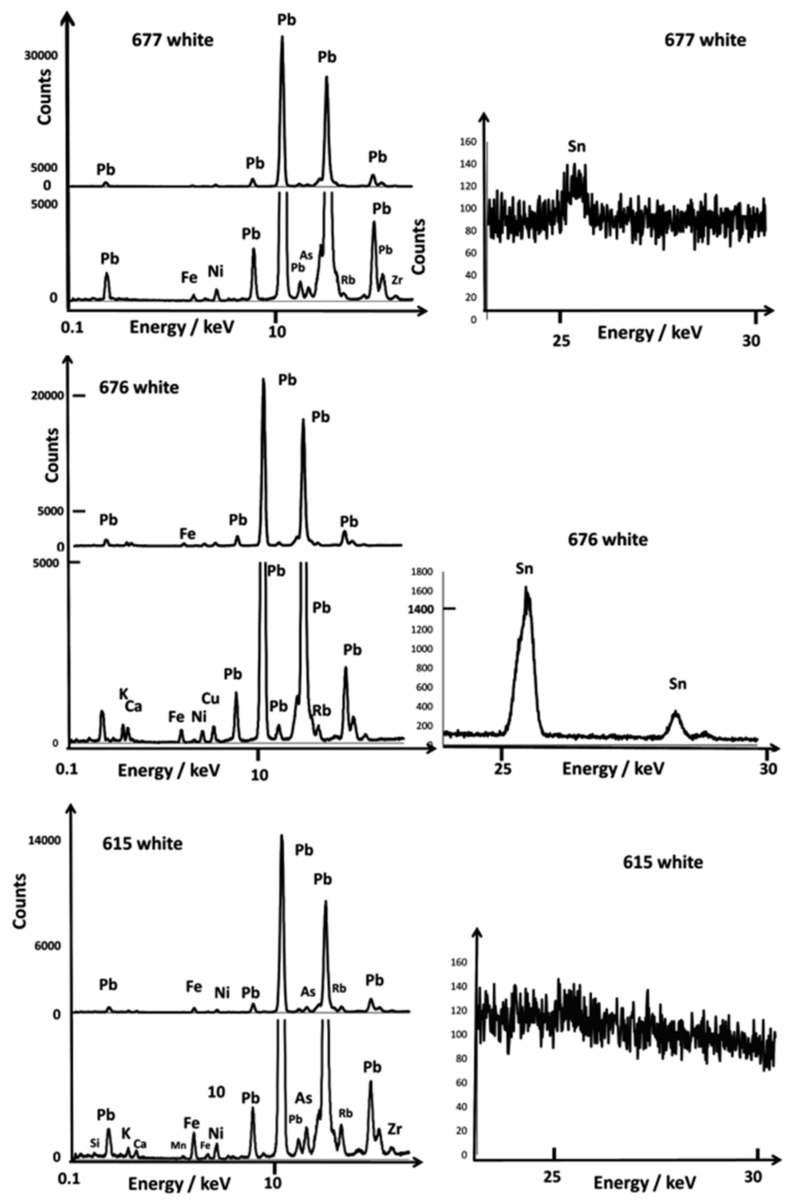
Comparison of representative XRF spectra for white painted decor in the 0.1–20 and 20–30 keV energy ranges.

**Figure 8:**
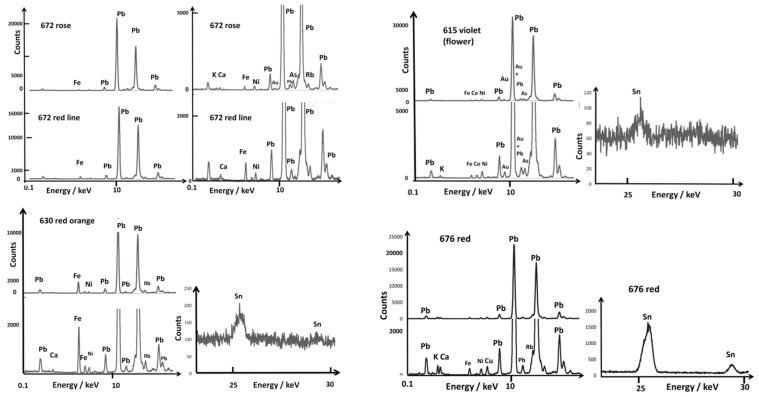
Comparison of representative XRF spectra for rose, red, and violet colors recorded in the 0.1–20 and 20–30 keV energy ranges.

**Figure 9:**
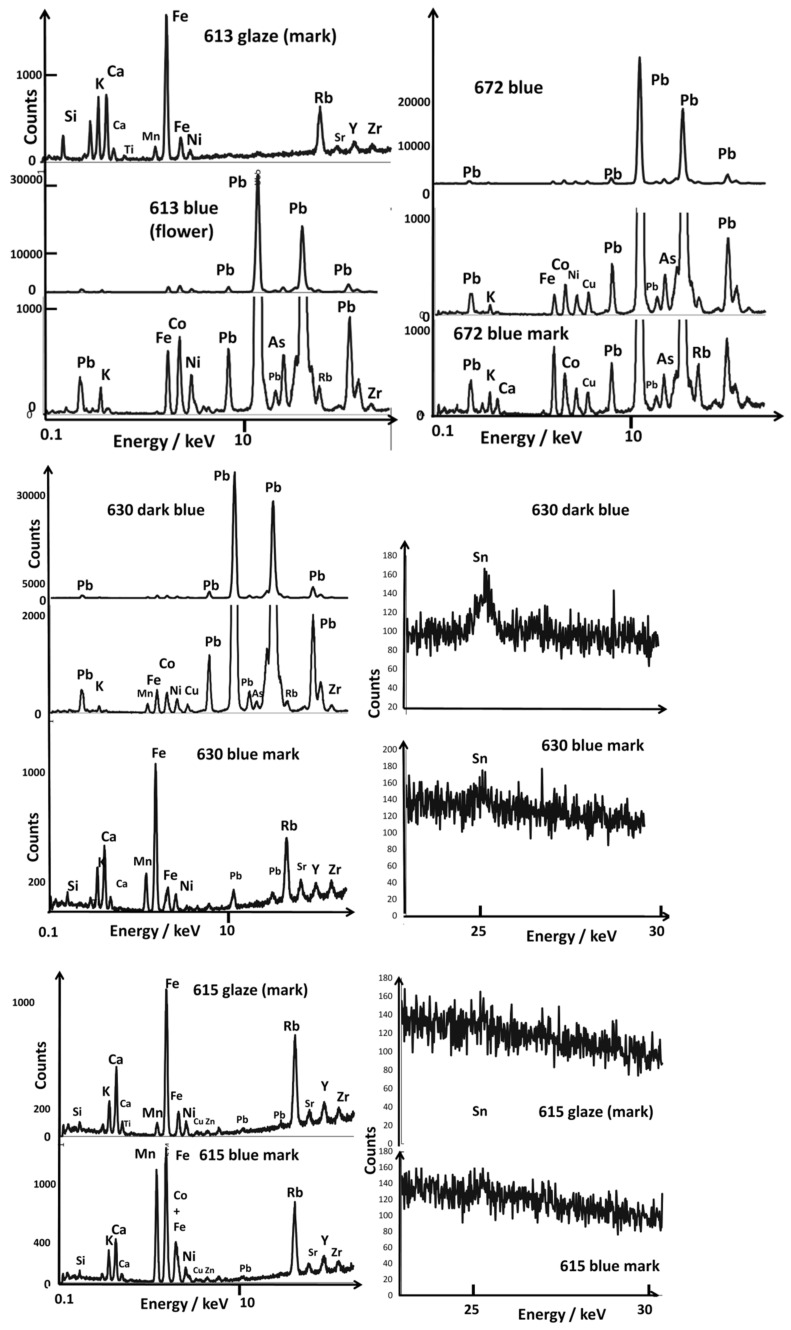
Comparison of representative XRF spectra for blue areas in the 0.1–20 and 20–30 keV energy ranges; for comparison, some spectra of paste and glaze coverage are also given; for comparison, glaze spectra are given.

**Figure 10:**
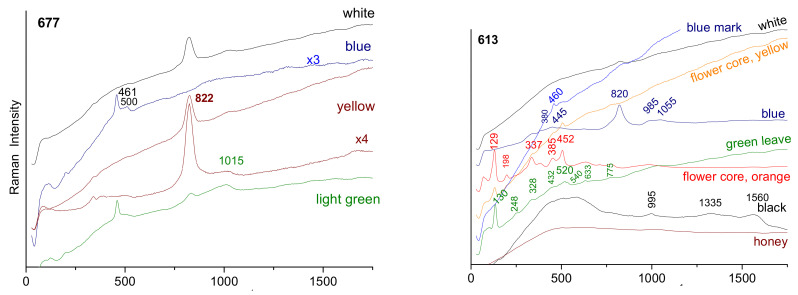

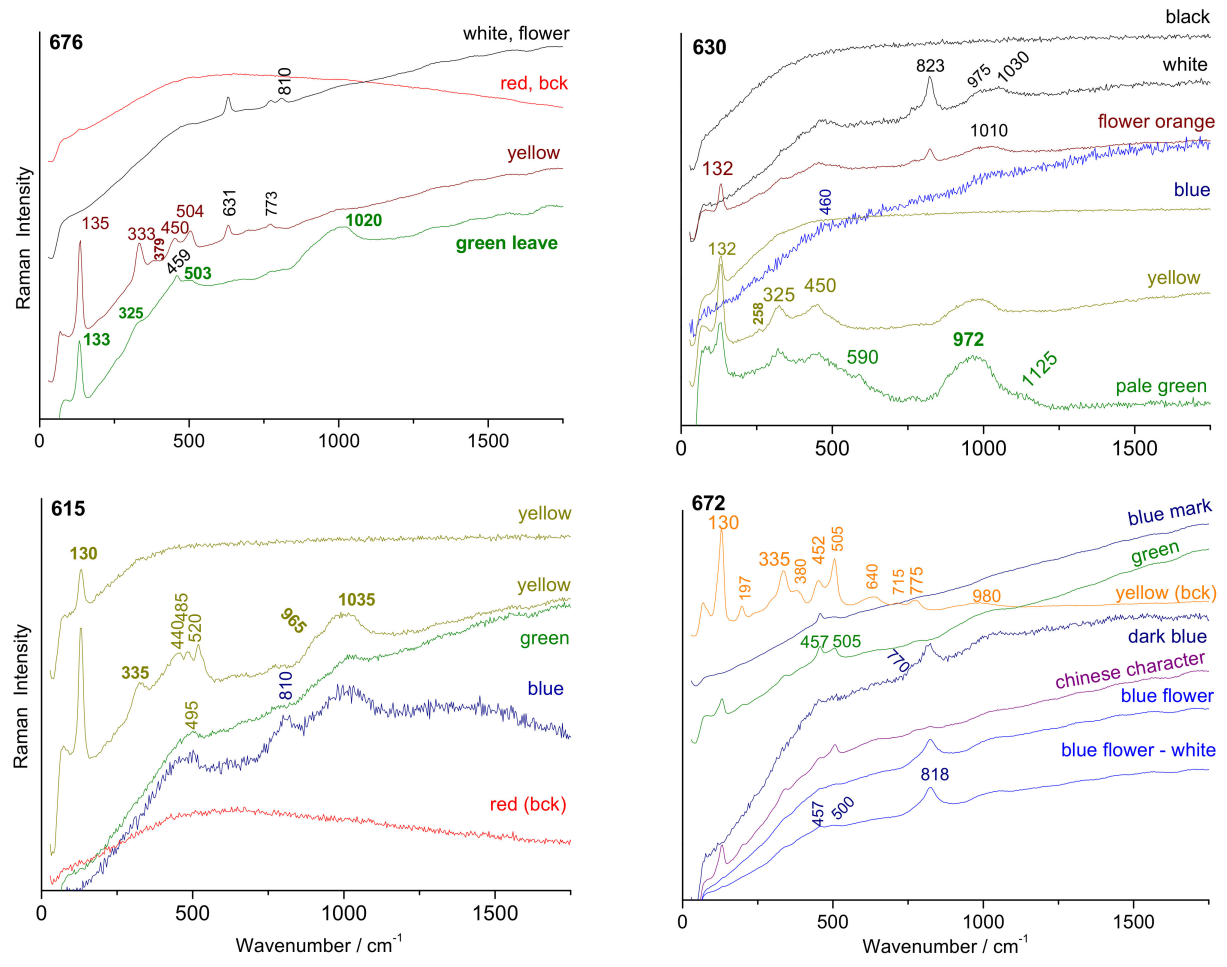
Comparison of representative Raman spectra for the different bowls.

**Figure 11:**
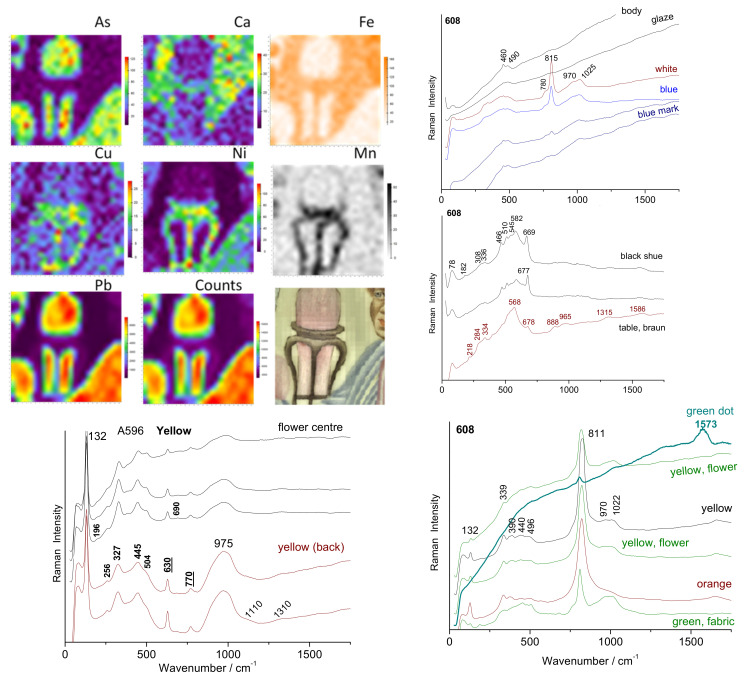
Comparison of representative Raman spectra for the different colored areas of two dishes (A596 and A608). XRF mapping of A608 center area for different elements showing the variation in their concentrations.

Figure 5, Figure 6, Figure 7, Figure 8 and Figure 9 present the representative XRF spectra of the different regions of ceramics (paste, glaze, and enamels), and Figure 10 and Figure 11 show the corresponding Raman spectra. Traces of lead due to the pollution of the surface of the body (e.g., A615, Figure 5) or the glaze (e.g., A615 mark, Figure 8) were detected between 10 and 15 keV. Indeed, the high volatility of lead oxide led to condensation on cooling at the whole surface of all artifacts in the kiln. The spectrum of glaze (e.g., A615), measured next to the mark, is also quite similar to that of the paste, except for the higher amount of potassium and lower iron, certainly due to some contribution of the glaze–paste interlayer.

We will focus more particularly on coloring agents, transition metals such as cobalt (blue color) and copper (green or red color if in the form of metallic nanoparticles, Cu°), as well as the other transition metals found as their impurities (manganese, nickel, and zinc) [50]. The contribution of the elements to the color is very variable. The power of coloration of cobalt is very strong (0.1 %wt CoO efficiently colors a silicate-based material), although 5 %wt MnO or Fe_2_O_3_ does not contribute when the firing is under a reducing atmosphere [50]. For the resolution of the instrument, the Kα peak of cobalt was confused (visually) with the Kβ peak of iron, although peaks of other elements were well observed. We will also consider the presence of arsenic, an element associated with cobalt found in hydrothermal deposits, such as those exploited in Europe [50]. Lead arsenates are very good opacifiers in the glaze and are very easily identified by Raman microspectroscopy [44,45,46,50]. The Kα peak of arsenic appearing confused with the representative peak of lead (Lα), only the Kβ peak is visible, just at the foot of the second strong peak of lead (Lβ) after a weak supplementary Pb peak, for example, on the spectra of bowl A613 (yellow spot) in Figure 6 and the white area of A615 and A677 in Figure 7. The peaks of bismuth are very close to those of lead and, therefore, difficult to identify visually. Only the calculation by simulation-optimization makes it possible to measure the number of photons due to this element, but it is important for the reliability of the comparisons that the elements used for the simulation of the spectrum be detected without ambiguity. Examination of the 20–30 keV spectral window informs very well about tin and antimony content (Figure 5). Note that the Fe peak is already rather strong for the body spectrum.

### 3.1. Glazed Background

The spectra of the yellow (A672 and A630), golden honey (A613), blue (A677 and A616), and red (A615 and A676) glazed backgrounds are shown in Figure 5. In all of the spectra, except that of A613 (golden honey), the peaks of lead are dominant. The high intensity of lead oxide peaks thus confirms its use as a fluxing agent for the bowl decoration. The honey color—or matte yellow—is actually obtained with gold containing small amounts of silver, nickel, mercury, and copper (Figure 5). The consideration of the ternary diagrams constructed from the net number of XRF photons characteristic of the different elements by simulating the spectrum will make it possible in the next paragraphs to compare the coloring technique and associated elements contributing either to the opacification or the shade or typical of the raw materials.

The red background of the A676 bowl shows the Cu peak a little stronger than the Fe one, which is consistent with the red color obtained by copper nanoparticles Cu°, a traditional Chinese technique [5,8,68]. The blue background (A616) exhibits a Co peak stronger than the Fe Kα peak, indicating coloration with a high level of Co^2+^ ions. Traces of tin are also observed. Yellow backgrounds (A630 and A672) exhibit mainly lead signature in addition to tin and antimony for A672 and only tin for A630. All backgrounds are, thus, different. Surprisingly, a tin signature was recorded for the red background (A676). The yellow backside of the A608 dish will be discussed further.

### 3.2. Painted Decor

Figure 6, Figure 7, Figure 8 and Figure 9 show representative XRF spectra of the painted decors. The green color is obtained by adding copper, Cu^2+^ ions being a traditional coloring agent [5,8] (spectra not shown). Figure 6 confirms that all of the yellow colors (A613 and A672) contain antimony and tin; only tin was measured in the A615 bowl. The level of tin in the green decor of A613 is significant in comparison with A630 and A676. Some white enamels are opacified with a phase containing lead and arsenic (A613, A615, A630, A672, Figure 7), but tin was detected at a high level in the A676 artifact and in A677 at a moderate level in association with arsenic, an unexpected mixture.

Rose (A672) and violet (A615) colors show XRF peaks of gold with traces of tin (Figure 8). The orange-red of A630 is obtained with iron, although the red of A676 shows the Cu peak stronger than the Fe one, which is consistent with a coloring by copper nanoparticles. The slightly higher level of Fe in the A672 red line suggests the use of this element to produce a brownish red hue, but a (very) small Au peak is also observed, as for the A672 rose. Black areas/lines also contain copper, iron, and manganese as well as traces of tin and antimony.

### 3.3. Reign Marks

Reign marks (Figure 2, Figure 3 and Figure 9), by design, usually feature the name of a specific reign period and then, from the Ming Dynasty onward, also the name of the dynasty. Kangxi-period imperial bowls bearing the Kangxi *yu zhi* mark (康熙御制) were, when first published, the subject of controversy over their authenticity.

In an article published in 1969 by Harry Garner [58], all of these marks were given as false based on the Jesuit archives uncovered a few years earlier by George Loehr [69]. Since then, Henry Garner’s opinion has been largely disproved, and the latest Foundation catalogs have revised these attributions [60]. The study of marks and their authenticity are the subject of research [53,54,70], but the study of their composition had not been undertaken in a global manner before our previous preliminary study [52]. Some blue marks appear visually to be made in overglaze (A608, A616, A630, and A672). From Figure 9, it is obvious that different types of cobalt sources are used for the imprinting of the marks: cobalt rich in arsenic for bowl A613 (Kangxi reign), cobalt rich in manganese for bowl A615 (Yongzheng reign), cobalt with copper (A616, Yongzheng reign), and cobalt rich in arsenic and copper (A672, Kangxi reign). Thus, we have a variety of cobalt sources/mixtures. Unexpectedly, traces of tin were observed for all blues.

### 3.4. Phase Identification

By identifying the phases, crystalline or amorphous, the Raman analysis makes it possible to go further in the identification of the enameling technique and coloring agents as well as the conditions of the decorations (see, e.g., references [44,45,46,50,51,52,55,57]). Remember that, due to the optics used, the volume analyzed is controlled at the micron scale, i.e., much smaller than the one probed by X-ray fluorescence. Therefore, there is no contribution from the underlying materials, the volume probed by Raman microspectroscopy being at the very surface (about 5 to 10 µm in depth, according to the focus).

The spectra of the blue-colored areas of bowl A630 (Figure 10, flower) are similar to those recorded on the blue marks (A613, A672, A615), with a characteristic bending peak of quartz (456 cm^−1^) and glassy phase (peak from 495 to 502 cm^−1^) of a glaze fired at higher temperature with the porcelain paste, as commonly observed for glazed porcelain [45,46,66,71,72,73]. The coloring is thus obtained by dissolving the cobalt ions (Co^2+^) in the lead-free glaze, which is a traditional technique [5,50,72]. The very large Raman cross-section of the As-O bond means that a small amount of lead arsenate induces a strong peak around 810–820 cm^−1^ (Figure 10), depending on the structure and composition of the lead (calcium/potassium) arsenate formed [50,66,67,73,74]. Table 2 summarizes the identified phases.

According to pXRF measurements, this arsenate signature is, therefore, observed for many enamels, in particular, white and blue, but not for most greens and yellows. Arsenic-rich blue decorations are identified not only in Imperial productions but also in many *Famille rose* porcelains [44,45,74,75,76,77,78]. As already established [49,52,53,71,72], enamels colored by metallic nanoparticles, gold or copper, give no or a very weak Raman signal but a characteristic broad fluorescence signal (e.g., for A613 honey, A676 red, A615 red); without the XRF spectrum, the nature of the metal cannot be clearly specified.

The two main types of yellow pigment, mainly lead-tin (spectrum ~132–325–350 cm^−1^ as for the yellow of A630) and complex pyrochlore based on antimony and zinc, usually called Naples yellow (yellow (bck) of A672 with, in particular, the components at 450 and 505 cm^−1^ plus eventually at ~200 cm^−1^), are identified in accordance with the previous works [55,77,78,79,80,81,82,83,84,85].

The complexity of the mixture used to achieve the hue is reflected by the presence of a small signal of pyrochlore yellow (~130 cm^−1^) in the red background of fluorescence of the metallic nanoparticles of A676 or the presence of arsenate (820 cm^−1^) in the light green from A677.

Enamels rich in lead present a wide band (SiO_4_ stretching band [86,87]) with several components around 980 cm^−1^ whose center of gravity shifts toward 1035 cm^−1^ when the lead content decreases. For the lead-free glaze, the mode is of very low intensity between 1000 and 1150 cm^−1^, as observed for the colorless glaze close to the marks (A672, A630, A613).

The presence of cassiterite (characteristic doublet at 633–775 cm^−1^ [44,46,51,55,66,67,88]) is clear for the yellow and white of the A676 bowl (Figure 10) and A608 and A596 dishes (Figure 11) and in traces for the soft green leaves of A613 and A672. This gives a particular character to these pieces, opacification with cassiterite being a European technique used for a few rare objects at the end of the reign of Kangxi or later [44,55].

Despite the low thickness of the enamels on the two dishes (A608 and A596), good Raman spectra were recorded, which indicates a prior preparation of well-crystallized pigments (Figure 11). A pXRF mapping carried out on site was possible due to the very good flatness of the object. The analysis shows a good agreement with the Raman spectra. The brown-black pigment is made of one (or several) phase(s) with iron-rich spinel structure (characteristic peak at 700 cm^−1^ [72,76,89,90]) and a manganese-rich phase (peak at ~570–580 cm^−1^ [90]). The manganese-rich phase is predominant for the brown color, with the addition of hematite, detectable by the component at 1315 cm^−1^ [90]. Traces of carbon are visible (doublet 1350–1585 cm^−1^). The pXRF mapping highlights the enamels painted on the glaze. The absence of iron and manganese (characteristic of Asian cobalt) in the blue of the man’s coat is evident. The cobalt is, therefore, imported from Europe [50,75].

## 4. Discussion

As noted in the Method section, pXRF examination from the top of the artifact cannot allow calculation of the composition. Firstly, the sodium signal will not be detected, due to the lack of a vacuum atmosphere during the measurement. The in-depth analysis explored by the instrument changes with the energy of photons, and, hence, the very top surface (a few microns) is explored for the light elements (e.g., Al, Si, K, Ca) and in-depth, more than a few mm, for heavy elements, such as tin or antimony [52,91]. Even for transition metals, it is possible that the probed thickness (up to ~200 to 300 µm) is greater than the thickness of the colored layer of enamel and, therefore, distorted by the contribution of the underlying glaze or body. However, the thickness of the enamel layer being similar to the depth probed by XRF for transition metals, a comparison of these elements’ ratios can be considered as reliable. Therefore, it makes no sense to want to determine an ‘enamel composition’, especially since the concentration of the coloring agent varies from point to point in the three directions for the creation of complex decoration. Thus, it will be necessary to evaluate the disruption of the XRF measurement of an overglaze by comparing the glaze/silicate matrix’s estimated contribution on a case-by-case basis. However, comparison of ternary diagrams calculated from the net number of XRF photons characteristic of the different elements offers a tool to compare elemental ratio and, hence, raw materials [52]. The results will be compared to the phase identification obtained by Raman microscopy.

The above-described procedure allows the comparison of the ‘local composition ratios’ of some colored areas of different objects, even when the measurements are made with different instruments. In fact, the constitution of ternary diagrams from the net number of XRF photons deforms the representation compared to what a ternary diagram calculated from the compositions would give. It is similar to the transformation of a geographical map incurred by replacing distance by travel time; the representation is distorted, but it is possible to compare and, in particular, to see whether the distribution of the data is spread out or clustered, defining groups.

### 4.1. Flux and Former

**Figure 12:**
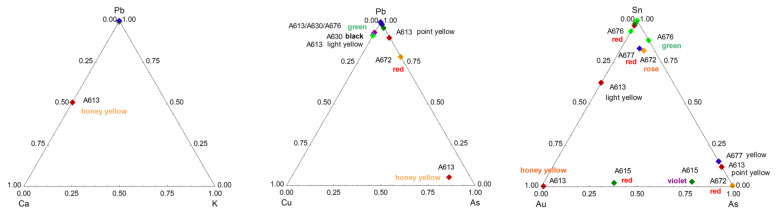

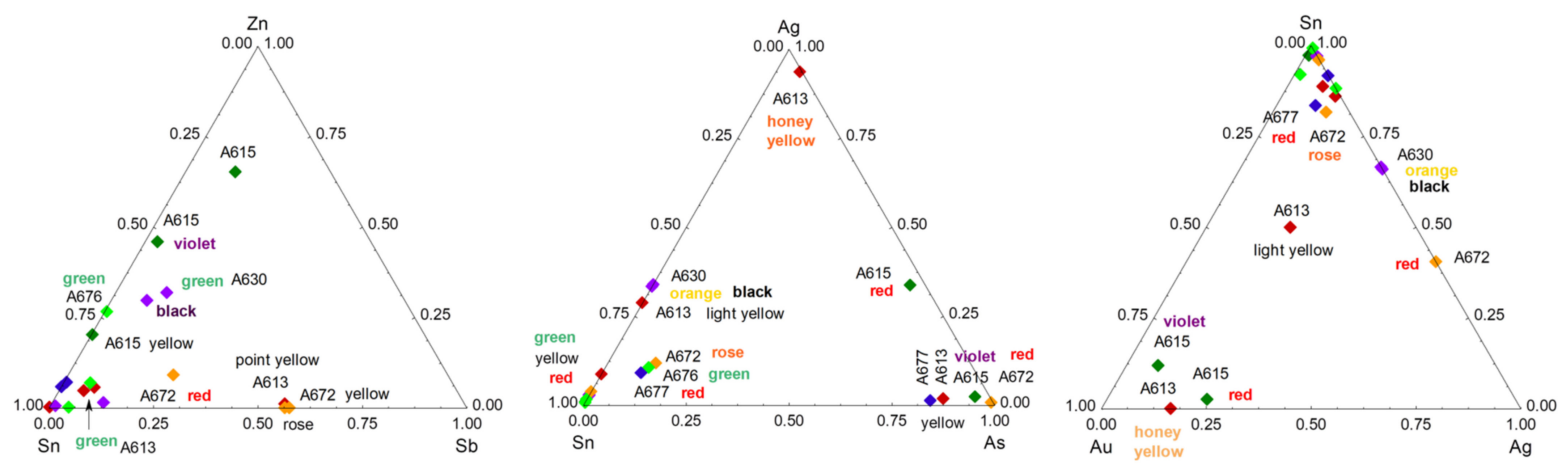
Comparison of the net number of photons of the elements Pb, Ca, K, Cu, As, Au, Sn, Ag, Sb, and Zn measured on the colored areas. Objects with special characteristics are indicated (see Table 1). Different colored lozenges are used to present the different objects (A677 blue, A613 dark red, etc.). The color indicated in the plots corresponds to that of the studied area (**red**, **violet**, **honey yellow**, **orange**, **green**, **rose**, yellow, and **black**).

The Pb-K-Ca ternary diagram (Figure 12) shows that all of the decoration is rich in lead, with the exception of the honey-gold background, which is also rich in calcium. This indicates that the gold is placed directly on the glaze and that the lead oxide is only a complementary flux. The very small thickness of gold particles usually measured on similar ceramics (typically 1 µm thick [45,76]) makes the thickness of the associated glassy matrix also thin, and, thus, the contribution of the underglaze layer will be dominant in the pXRF spectrum. The significant signal of arsenic should be noted. Arsenic is usually added to obtain a good sticking between the gold particles (thickness ~1 µm) and the ceramic substrate [92,93]. The Pb-Cu-As ternary diagram confirms that all of the decoration is rich in lead, with the exception of the A613. The Sn-Au-As ternary diagram shows the decoration (rather) rich in arsenic (A615 red and violet, A672 red, A613 and A677 yellow). Sn-Zn-S, Sn-As-Ag, and Sn-Au-Ag ternary diagrams confirm the presence of Sn in many enamels.

**Figure 13:**
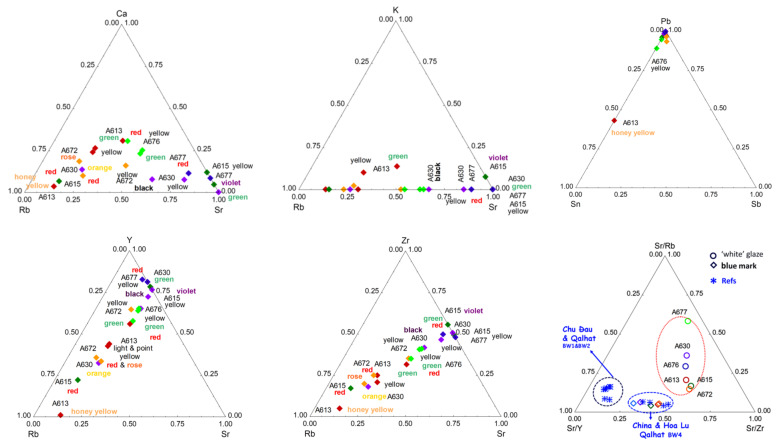
Comparison of the net number of photons of the major (Ca, K) and related trace elements (Y, Rb, Sr, Zr) measured on the colored areas. The data of white and enamel/glaze analyses are plotted separately and compared with the previous data of Chinese and Vietnamese (blue star) artifacts excavated on the kiln site and other places [96,97,98,99]. Objects with special characteristics are indicated (see Table 1). See Figure 12 for a labeling explanation.

The comparison of the impurity diagrams (Y-Rb-Sr and Zr-Rb-Sr, Figure 13) shows the analysis points aligned according to a constant Y/Sr and Zr/Sr ratio, respectively, which indicates that these elements are provided by the same raw material. Data are also rather well-aligned on the Rb-Sr-Ca diagram, except for some enamel that appears Rb-free but Sr-rich. It is usual for the zirconium to be present in the form of zircon partially substituted by yttrium. Very stable, zircon is present in igneous rocks and is preserved in sands and detrital rocks and in enamels made with these raw materials.

### 4.2. Gilding Technique

The Sn-Au-As (Figure 12) and Sn-Sb-Pb (Figure 13) ternary diagrams highlight the use of gold not only for the honey background of A613 but also for the red background of A615. The high level of the arsenic signal is consistent with a preparation of colloidal gold following Perrot’s recipe (and not Kunckel’s method based on the use of Sn to prepare colloidal gold) [67,94], as observed for many European red enamels of the 17th and 18th centuries. It is possible that the dull shine resulted from the craftsmen’s ignorance of the need to polish the golden surface with agate to obtain the orientation of the gold particles and, thus, the brilliance. The low intensity of the lead signal is also related to the thinness of the gilding—and that of the associated amorphous ‘enamel’ matrix—that leads to an important contribution of the underlying substrate. The signal of silver is well correlated with that of gold. Indeed, silver is generally alloyed with gold to promote bonding with the silicate substrate [92]. The detection of gold in white areas of the A613 artifact may indicate that the gold background is put on the whole surface, and, hence, there is a contribution of the underlayer with gold. The detection of mercury indicates the application of a cold gilding. This may be due to contamination during the restoration with gold lacquer (kintsukuroi 金繕い) or an attempt to improve the appearance of the gilding.

### 4.3. Yellow, Green, and Red to Pink Colors

**Figure 14:**
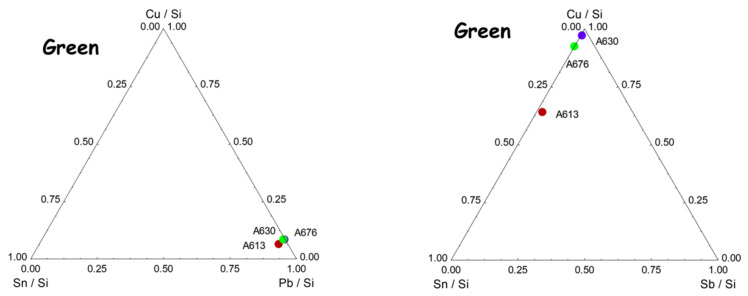
Comparison of the net number of photons of the elements Cu, Sn, and Pb (**left**) and Cu, Sn, and Sb (**right**) measured in the green areas. Data are normalized by Si signal.

Copper (Co^2+^ ion) is used as a green chromophore (Figure 14). The presence of zinc in the green and yellow-green pyrochlore pigments already observed for the pigments of French enamels [56] and for other Chinese enamels [55] is confirmed. On the contrary, antimony is mixed with tin only for some yellow (the A672 and A613 bowls, both assigned to the Kangxi period), as previously observed [5,8,55]. However, tin is detected in many colored areas: red, rose, orange, green, yellow, and even in some black decors, likely due to the contribution of the sub-layer on which the black was put. In most cases, no cassiterite formation is observed with Raman scattering (except for A676 and 608 artifacts), which suggests that tin was brought as an impurity of one of the elements, probably lead. The red background of A676 obtained by the metallic Cu° precipitates requires a control of the redox reactions by multivalent ions such as Sn and Fe, which are effectively observed. This process has been used since Roman times [43,68,95].

**Figure 15:**
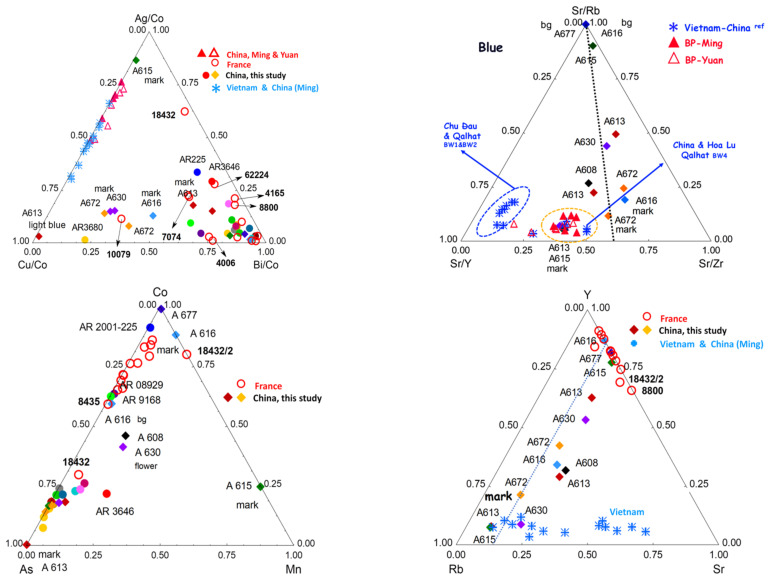
Comparison of the net number of photons of the major, minor, and trace elements measured on the blue-colored areas (rhomboids for Baur Foundation collection (A) and solid circles for Ariana collection (AR) published in [52]). Samples labeled with AR are porcelains from Jingdezhen kilns enameled at Guangzhou or Jingdezhen [52]. Reference data (open and solid red triangle referring to Yuan and Ming artifacts [96,97,98], open red circle to French soft-paste porcelain [66], and blue star referring to Vietnamese and Chinese artifacts [96,99]) are added for comparison. Objects with special characteristics are indicated (see Table 1). See references for artifact photos; inventory numbers are indicated for artifacts located at the cluster border. Circles and lozenges of various colors, this study, see Figure 12 caption.

The comparison with the Sr-Y/Zr-Rb data (Figure 13) characteristic of the silicate matrix of enamels/glazes and concerning Chinese blue-and-white porcelains attributed to the Yuan and Ming periods [96,97,98,99], but also contemporary Vietnamese productions from the same periods [99], distinguishes very well the different origins, which is consistent with the use of different raw materials (clays, sand, feldspars, etc.) for each group concerned. Remember that the Sr and Rb elements are flux impurities (K, Ca, Na), while Y and Zr are impurities in refractory materials bringing aluminum and silicon, forming the silicate network. These elements are, thus, very characteristic of the raw materials used to prepare glaze and enamels. It is noted that, for the studied objects, certain ratios are constant but that the Rb content is very variable, which indicates that the quantity of the material providing this element varies greatly. The distribution of the data on a linear axis, which corresponds, in the case of Figure 13 and Figure 15, to constant Y/Sr and Zr/Sr ratios, characterizes the use of varied proportions of different raw materials. This can also come from the variability in the thickness of the enamel layer and variable contribution of the material of the substrate.

### 4.4. Blue

The cobalt-rich raw material used by potters during the Ming period and most of the Kangxi reign is derived from primary Asian geological sites [50]. It contains an equivalent quantity of other transition metals (iron, manganese, etc.), which imposes a firing under strict reducing conditions to obtain a correct blue color. However, an oxidizing atmosphere leads to ‘dirty’, blackish, or greenish hues and black spots [50]. On the contrary, European (and Persian) cobalt ores are mined from secondary geological sites, hydrothermal veins rich in arsenic, sulfur, nickel, bismuth, copper, and silver [50]. The Co-As-Mn, and Ag-Cu-Bi diagrams (Figure 15) classify very well the different raw materials contributing to the blue color (uranium is also an important element, and more data concerning this element should be collected). Only the A615 mark is typical of Ming cobalt, due to its high level of Mn [96,97,98,99,100,101,102,103,104,105,106,107,108,109,110,111,112,113]. The cobalts of the A616 mark and background of the A677 bowl are very pure and, therefore, indicate in no way a production of the 18th century [44]. The purity of the cobalt of the A676 mark, much purer than for the areas of enamel of the same color, may indicate that the mark was affixed later, in the 19th century. The arsenic-rich blues certainly use cobalts imported from Europe, not only characterized by the significant level of arsenic but also of bismuth/copper, silver, and nickel. Those containing both arsenic and manganese elements are mixtures of different coloring raw materials.

## 5. Conclusions

The technical solutions appear very different from one object to another, which leads one to think that each bowl is really a unique object and not an item produced in (small) series. Two categories of reign marks are highlighted. The A615 and A613 underglaze blue marks are covered with a K-rich, lead-free, or lead-poor glaze. This definitively supports the deposition of these marks on the body before firing the body and glazing at Jingdezhen (underglaze mark) for these two artifacts. The other marks are overglazed. They could have been applied with overglazed decoration firing or afterwards, by a special firing at a lower temperature. Examination of Mn, Ag, Ni, Sn, Sb, and As (U not shown on figures) signals in the blue areas show the presence of different groups. The A677, A616, A615, and A613 marks are arsenic-free, and another group is arsenic-poor (A608 (dish) and A630 (19th century)). The A615 bowl mark is rich in Mn, as observed in artifacts produced during the Ming Dynasty. A ‘return’ to traditional Chinese techniques has already been observed for Yongzheng productions [77].

The cobalt of the A677 decoration (attributed with reserves to the reign of Kangxi) and the A616 mark (attributed to the reign of Yongzheng, also with reserves) are quite pure, free of/poor in manganese, and almost free of As, Ni, and Fe. Such a level of purity is strange, and the assignment of artifacts to the period of the reign mark must be questioned. Assignment of the A677 decoration made after 1850 is, thus, consistent with the analytical data. The mark of the A616 bowl also uses rather pure cobalt, but some other characteristics come close to those ascertained in the main group. The question now arises: is that consistent with the addition of the overglazed mark perhaps made on the bowl many years after its production? It is important to note that data measured on blue areas of blue-and-white French soft-paste porcelains from the 17th and early 18th centuries [66] and of enameled watches from the same period [56] are located in the same cluster as Imperial and Guangzhou wares in the Ag-Cu-Bi (Co-normalized) ternary diagram (Figure 15). This definitively supports the use of imported cobalt. The observation of the ternary Y-Rb-Sr, impurities characteristic of the raw materials used to produce the silicate matrix of the enamel, shows that the enameled objects in France form a very different cluster from the enameled objects in Guangzhou or Beijing [52], except the A615, A616, and A677. Therefore, for the other objects, only the coloring matter was imported from Europe, while it is probable that for the three objects belonging to the same cluster, the complete enamel powder was imported. Bowls A613 and A677 bear the marks of the reign of Kangxi and A616 of Yongzheng.

It clearly appears that the blue areas of the objects studied contain more arsenic than the European objects; this indicates either a difference in the degree of selection/purification of the cobalt source (several grades are mentioned in 18th century documents, as reported in [50]) or a deliberate addition.

Objects A608 and A630 have a similar and higher Mn/Fe ratio and are attributed to the Qianlong period; this higher Mn level is consistent with the use of a mixture of European and Asian cobalt to reduce the cost of production. The mixing of different sources, either optimizing the hue or reducing the cost, has already been reported (see in [50]); we also observe a lower As content, as expected for such a mixture. On the other hand, the blue areas of A630 (Daoguang), A608 (Qianlong dish), A616 (uncertain Yongzheng), and A677 (uncertain Kangxi) are in the same group as the enamels attributed to the Guangzhou workshops.

**Figure 16:**
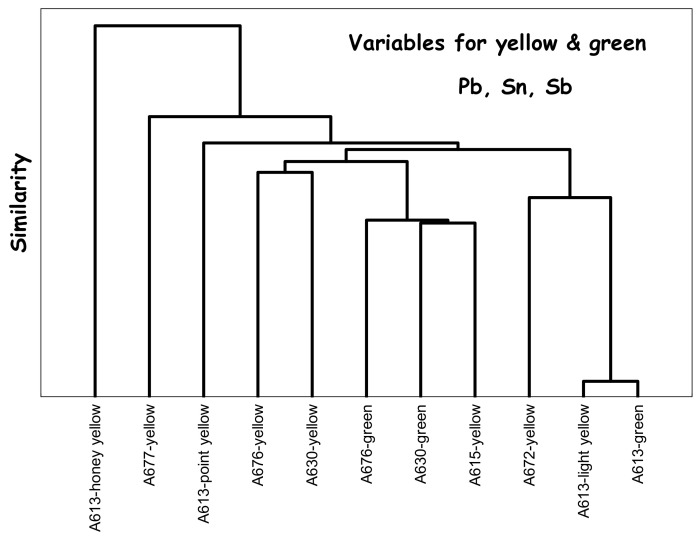
Euclidian dendrogram drawn for yellow and green colors.

The background of Au° red (A615, Yongzheng) and the other of Cu° red (A676, mark and perhaps period of Kangxi) show a back-and-forth between European and Chinese recipes. For spear opacification, the use of cassiterite is exceptional (A676). The dendrogram constructed for the yellow and green colors colored by Naples yellow pyrochlore-type pigments (Figure 16) shows the variety of signatures. At least three different yellows, the classic Pb-Sn used since the Ming period but also Pb-Sn-Sb-(Zn) complex pyrochlores with different Sn/Sb/Zn ratios. One bowl, A677, clearly appears to be a 19th century ‘copy’; another probably had the addition of a reign mark after its manufacture (A616).

This work demonstrates the possibility of comparing the characteristics of composition and the phases formed in a non-invasive way. The comparisons of the distributions of the normalized XRF count numbers give a representative statistical view, despite the different variabilities intrinsic to the sophistication of the decorations. The recognition of specificities requires the study of series of comparable parts.

## Figures and Tables

**Table 1:** Studied artifacts and their characteristics (inventory numbers are simplified in the text using A followed by the last three-digit number in bold); areas analyzed by XRF and Raman (underlined) spectroscopy are given (bck: background); colored spots only studied with Raman are in brackets and underlined.

Artifact	Inventory Number	Reign Mark	Dimension/cm and *Weight/g*	Spots Analyzed by XRF and Raman							
				Body	Glaze	Yellow (Honey)	Blue	Green	Red-Orange	White	Black
bowl	CB.CC.1936.**677**	Kangxi reign mark in red but probably later period	D. 14.5; H. 7.4; *297*		yes	yellow	bck		red	white	
bowl	CB.CC.1931.**676**	Kangxi mark in colloidal red	D. 14.5 H. 6, 1; *127*			yellow		green	red	white	
bowl	CB.CC.1932.**613b** (from a pair)	Kangxi mark in cobalt blue	D. 12.5; *151*	yes	close to flower close to mark	light-yellow honey-yellow	flower light mark	green		white	
bowl	CB.CC.1950.**672**	Kangxi reign mark in cobalt blue	D. 12.5; H. 6.5; *156*	yes	close to mark	yellow	flower mark		rose scale line	white	
bowl	CB.CC.1937.**615**	Yongzheng reign mark in underglaze cobalt blue	D. 11; *108*	yes	close to mark	yellow	flower mark		red(bck) violet(scale)	white	
bowl	CB.CC.1930.**616**	Yongzheng reign mark but maybe later date	D. 9.3; *74*		close to mark		-blue (bck) -mark				
dish	CB.CC.1936.**596**	Yongzheng reign mark in overglaze cobalt blue	D. 20			(yellow)					
dish	CB.CC.1935.**608**	Qianlong reign mark in cobalt blue	D. 17.4; *169*				men coat (mapping)				
bowl	CB.CC.1930.**630**	Daoguang reign mark in cobalt blue	D.18.5; *489*		close to mark	yellow	flower mark	green	orange	white	black

**Table 2:** Phases and elements detected (major elements in bold, underlined; NY: Naples yellow (Pb_2_Sb_2−x_M_x_O_7-δ_); matrix: main Raman peaks of the silicate matrix in which the coloring agents are dispersed).

Artifact	Reign Mark	Phases and Characteristic Elements (Major, *Minor/Traces*; Main Raman Peaks (cm^−1^))								
		Background (Color)	Mark	Yellow	Blue	Green	Violet, Red to Orange	White	Black	Matrix
Bowl A677	Kangxi reign mark in red but probably later period	(blue: Co^2+^) Quartz, glassy phase. **Co**		Arsenate (822) **Pb,As**,*Sn*	Bck Mn,Fe,Co, Ni,Cu,Zn	Arsenate (822) Quartz Glassy phase *NY*	Fe,Ni,Sn	Arsenate (822) **Pb**,*As,Sn,Fe*		(1015)
Bowl A676	Kangxi mark in colloidal red	(red: Cu) **Pb**, *Sn,Cu,Ni* Fluorescence		Cassiterite NY (135,333, 505) **Pb**,*Sn,Sb* *As,Cu,Ni,* *Fe*		NY (133,325,459)	Red: **Pb,Sn** *Fe,Ni,Cu,*	Cassiterite *Arsenate (810)* **Pb, Sn,** *As,Cu,Ni,* *Fe*		
Bowl A613	Kangxi reign mark in cobalt blue	(honey:Au) **Au**,**Ag**,Hg, *Pb,Sn,As* Fluorescence	Quartz, glassy phase *Mn,Fe,Ni,* *Co*	Light yellow: Sn,Cu NY (129,198,452) **Pb,***As,Fe,* *Ni,Cu,Sn,* *Sb*	Arsenate (820) **Pb,***As* *Co, Fe,Ni*	NY (130,328,520) **Pb,Cu,***As,* *Ni, Sn* *(trace of cassiterite)*	Violet: **Pb**,Au,*Fe,* *Ni,Co,As,* *Sn*	Arsenate (820) **Pb, Au,***Cu*,*As,* *Fe,Ni, Sn*	580	1020
Bowl A672	Kangxi reign mark in cobalt blue	(yellow) **Pb**, *Sn,Sb*	Quartz, glassy phase	NY (130,197, 335,505) **Pb,***Fe,Ni, Sn*,Sb	Arsenate (818) **Pb,***As,Fe,* *Co,Ni*	Cassiterite trace? NY (130,197,335, 505)	Rose: **Pb**,*As,Au,* *Sn,Fe,Ni* Red: **Pb**,*Fe,Ni*	**Pb**,*As,Fe,Ni, Sn*,Sb		
Bowl A615	Yongzheng reign mark in cobalt blue	(red) **Pb**,Au, *Sn,Cu,Ni,As* Fluorescence	*Fe,Mn,Co,* *Ni,*	NY Arsenate (810) **Pb,Sn***,Ni,Zn*	Arsenate (810) **Pb,***As,Fe,* *Co,Ni, Cu,Mn,Sn*	Glassy phase **Pb,*****Sn****,Cu,* *Ni,Zn,Mn*	Violet (scale) As,Au,*Zn*	**Pb, As** *,Fe, Ni*		1035
Bowl A616	Yongzheng reign mark but maybe later date	(blue) **Pb**,*Sn,Co,* *Mn,* *Fe,Ni,As*								
Dish A596	Yongzheng mark and period	(yellow) ^a^ Cassiterite NY (132,327,445)		Cassiterite NY (132,325,445)						975 (lead-rich)
Dish A608	Qianlong reign mark in overglaze cobalt blue		Quartz, Glassy phase Arsenate (780–815)	NY Arsenate (780–815)	Arsenate (780–815) *Co,Ni,As*	Arsenate (780–815)	Arsenate (780–815) NY(132)	Arsenate (780–815)	Spinel MnO_2_ **Pb**,Cu,Mn, Ni,Fe,As	970–1020 (lead-rich)
Bowl A630	Daoguang 1825–1850 *Fe,Mn,* *Ni,Co* *underglaze*	(yellow) **Pb**,*Sn,* *Fe,Ni*	*Fe,Mn,Ni,* *(Co)*	NY (132,325,450) **Pb,Sn***,Ni,* *Fe,Cu*	Quartz, Glassy phase **Pb,***As* *Co,Cu,Mn,* *Fe,Ni,Sn*	NY (132,325,450) **Pb,Sn***,Ni,* *Fe,Cu*,S**b**	(Orange) Arsenate (823) NY (132) **Pb**,*Fe,Ni,* *Sn*	Arsenate (823)	*Fluorescence* **Pb,Cu,***Fe,* *Ni,Mn,Sn*, S**b**	975–1030 (lead-rich)

^a^ backside.

## Data Availability

Not applicable.
